# Beta-escin has potent anti-allergic efficacy and reduces allergic airway inflammation

**DOI:** 10.1186/1471-2172-11-24

**Published:** 2010-05-21

**Authors:** Ines Lindner, Christiane Meier, Angelika Url, Hermann Unger, Andreas Grassauer, Eva Prieschl-Grassauer, Petra Doerfler

**Affiliations:** 1 Marinomed Biotechnologie GmbH, Veterinaerplatz 1, A-1210 Vienna, Austria; 2 Institute of Pathology, Department of Patholobiology, Veterinary University Vienna, Veterinaerplatz 1, A-1210 Vienna, Austria; 3 Laboratory of Tropical Veterinary Medicine, Veterinary University Vienna, Veterinaerplatz 1, A-1210 Vienna, Austria; 4 Veterinary University Vienna, Veterinaerplatz 1, A-1210 Vienna, Austria

## Abstract

**Background:**

Type I hypersensitivity is characterized by the overreaction of the immune system against otherwise innocuous substances. It manifests as allergic rhinitis, allergic conjunctivitis, allergic asthma or atopic dermatitis if mast cells are activated in the respective organs. In case of systemic mast cell activation, life-threatening anaphylaxis may occur. Currently, type I hypersensitivities are treated either with glucocorticoids, anti-histamines, or mast cell stabilizers. Although these drugs exert a strong anti-allergic effect, their long-term use may be problematic due to their side-effects.

**Results:**

In the course of a routine *in vitro *screening process, we identified beta-escin as a potentially anti-allergic compound. Here we tested beta-escin in two mouse models to confirm this anti-allergic effect *in vivo*. In a model of the early phase of allergic reactions, the murine passive cutaneous anaphylaxis model, beta-escin inhibited the effects of mast cell activation and degranulation in the skin and dose-dependently prevented the extravasation of fluids into the tissue. Beta-escin also significantly inhibited the late response after antigen challenge in a lung allergy model with ovalbumin-sensitized mice. Allergic airway inflammation was suppressed, which was exemplified by the reduction of leucocytes, eosinophils, IL-5 and IL-13 in the bronchoalveolar lavage fluid. Histopathological examinations further confirmed the reduced inflammation of the lung tissue. In both models, the inhibitory effect of beta-escin was comparable to the benchmark dexamethasone.

**Conclusions:**

We demonstrated in two independent murine models of type I hypersensitivity that beta-escin has potent anti-allergic properties. These results and the excellent safety profile of beta-escin suggest a therapeutic potential of this compound for a novel treatment of allergic diseases.

## Background

The prevalence of allergic diseases, such as allergic rhinitis, allergic conjunctivitis, allergic asthma, food allergies and anaphylaxis has constantly increased in the developed world during the last decades [1,2]. These diseases are caused by a dysfunction of the immune system in response to normally harmless environmental agents and are characterized by an excessive activation of mast cells, basophils and T helper cells of the Th2 linage. Type I hypersensitivity reactions involve many types of inflammatory cells, but mast cells and eosinophils are the two main effector cell types. The allergic inflammation is often classified in three temporal phases. In an immediate early phase allergic reaction, Immunoglobulin E (IgE) bound to FcεRI on mast cells and basophils is crosslinked by repeated allergen exposure. Consequently, these cells release various pre-formed or rapidly synthesized chemical mediators. These mediators, such as histamine, elicit vasodilation, increased vascular permeability, oedema and acute functional changes in affected organs. The IgE-mediated early phase reaction occurs within minutes of allergen exposure. It can be either localized with symptoms such as rhinitis, conjunctivitis, acute asthma attacks and hives, or systemic, resulting in a potentially lethal anaphylactic shock. The following late phase response is initiated by cytokines and chemokines derived from mast cells, resulting in recruitment of leukocytes into the affected tissues [2-5]. In the case of allergic asthma, for example, the observed clinical symptoms are airway oedema, increased mucus production, recurrent bronchospasm and sustained inflammation of the tissue by infiltrating leukocytes. Finally, it may result in a chronic allergic disease, including tissue damage and remodelling [6-9].

Type I allergies are commonly treated by corticosteroids, anti-histamines and mast cell stabilizers. These drugs are anti-inflammatory, or block the action of allergic mediators by preventing activation of cells or degranulation processes. Also, bronchodilators are used for the treatment of allergic asthma. All these therapeutics help to alleviate the symptoms of allergy but, especially after long-term and high-dose medication, they can have quite substantial side-effects. In particular, oral corticosteroids can cause general immuno-suppression, skin fragility and Cushing's syndrome, and even new generation anti-histamines are not completely void of sedative effects [10,11]. Therefore, there is still a vital need for the development of new anti-allergic drugs with satisfactory tolerability for long-term use. They should be effective on the early as well as the late phase allergic reactions.

In our laboratory, a series of natural extracts have been screened *in vitro *for their potential anti-inflammatory and anti-allergic effects. Among these extracts, low concentrations of the test substance beta-escin showed an inhibitory effect in IgE-antigen-stimulated mast cells. Beta-escin is the main active constituent of Aesculus hippocastanum seed extracts, which is composed of a mixture of triterpene saponins [12]. It is a well known substance with numerous pharmacologically interesting effects. Beta-escin was reported to possess anti-inflammatory activities, venotonic properties and endothelium-protectant effects [13] which were supported by different animal models [14-20]. Furthermore, several clinical studies demonstrated an excellent tolerability of beta-escin. Thus, beta-escin is widely used for the treatment of chronic venous insufficiency, post-operative ileus, haemorrhoids, acute impact injuries and is included in cosmetic products [13,21-24].

However, to date beta-escin has not been described to prevent or alleviate allergic diseases. In our study we investigated the anti-allergic efficacy of beta-escin in two different experimental mouse models of allergy: The passive cutaneous anaphylaxis (PCA) model is a widely used *in vivo *model to examine the anti-allergic effect of compounds on immediate hypersensitivity type I reactions because it mimics an acute mast cell-mediated reaction [25,26]. Ovalbumin (OVA)-induced allergic airway inflammation is a model which exhibits symptoms of acute allergic asthma. It is caused by a late phase allergic reaction and is characterized by the inflammation of airways and lung tissue [8,27]. Our results from these *in vivo *models show that beta-escin is a highly promising anti-allergic compound.

## Results

### Beta-escin significantly reduces the PCA reaction in mice

In the PCA model, skin mast cells are passively sensitized with antigen-specific IgE and activated by systemic administration of the corresponding antigen. This leads to a massive release of allergic mediators and the permeabilization of capillary blood vessels. If Evans blue dye is applied together with the antigen, this effect can be visualized by extravasation of the dye. The extension of the blue stained skin area is therefore taken as a measure for the intensity of the early phase of the hypersensitivity reaction.

**Figure 1:**
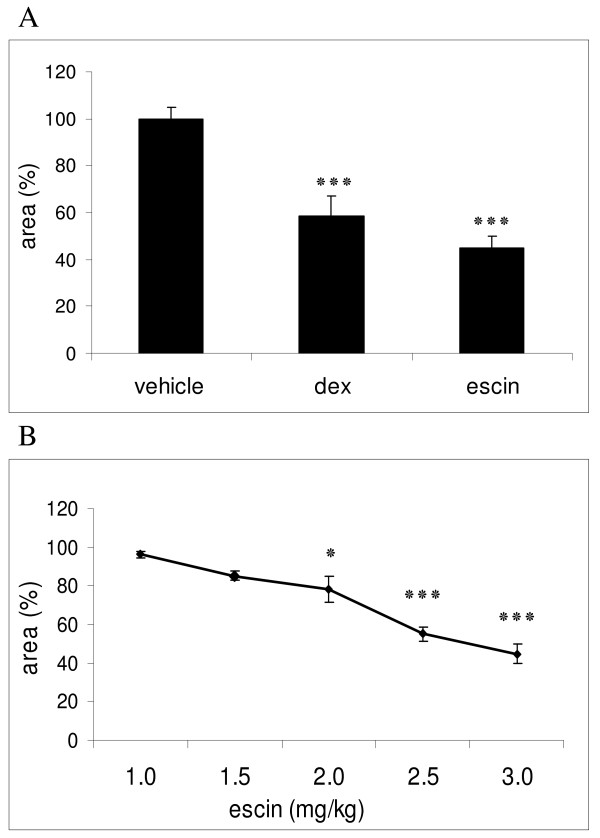
**Anti-allergic and dose-dependent efficacy of beta-escin in the murine PCA model**. For PCA induction, anti-TNP IgE (2.5 μg) was injected intradermally into the dorsal skin at 6 independent spots. After 15 min, mice were challenged with intravenous injection of 50 μg DNP-BSA supplemented with 1% Evans blue dye. Blue stained skin areas were quantified from digital photographs 15 min after challenge. (A) SKH1-mice were pre-treated i.p. with vehicle (10 ml/kg, n = 21), dexamethasone (dex, 3 mg/kg, n = 5) or beta-escin (escin, 3 mg/kg, n = 12) 6 h prior application of antigen. (B) Different concentrations of beta-escin (n = 4-12) were given i.p. 6 h prior challenge. For each mouse the mean of 6 spots was calculated. Based on these values the mean ± SEM was determined for each group, normalized to the vehicle control group (equals 100%) and represented as column or point in the graphs (A) and (B), respectively. Significant differences between the treatment groups and the vehicle group are indicated with: * p < 0.05 and *** p < 0.001.

To examine the anti-allergic effect of beta-escin *in vivo*, we first evaluated the substance in this PCA model. Mice of the negative control group, which received 0.9% sodium chloride solution (NaCl) or PBS, developed a strong PCA reaction (Figure 1A). Five minutes after challenge the spots of these vehicle-treated mice were already clearly visible and expanded during the next 10 min. The mean dimension of the blue spots was 38.8 mm^2 ^± 1.9 SEM at 15 min after antigen application, which was considered as 100% response. In the positive control group, mice were treated with 3 mg/kg dexamethasone, which is typically used as reference compound in allergic animal models [28]. The appearance of blue spots at the sensitized skin areas of dexamethasone-treated mice was delayed, and the areas were significantly smaller in comparison to the vehicle control group. At 15 min the blue spots measured only 58.3% compared to the vehicle control (Figure 1A). Strikingly, treatment with 3 mg/kg beta-escin also strongly suppressed the PCA reaction. The mean area of the blue spots at 15 min was limited to 17.4 mm^2 ^± 2.0 SEM, representing only 44.7% of the mean area from vehicle-treated animals (Figure 1A). This indicated that beta-escin inhibited the anaphylactic reaction more potently than dexamethasone. Considering the different molecular masses of both compounds, this efficacy of beta-escin is particularly remarkable. The molecular mass of beta-escin is 1137.3 g/mol, which is 2.8-fold higher than the molecular mass of dexamethasone (392.5 g/mol). Therefore, our results with 3 mg/kg of each compound suggest that beta-escin inhibits the PCA reaction more than 3-fold better than dexamethasone when comparing molar concentrations.

### The anti-allergic property of beta-escin is dose dependent

In the next experiment, we examined the dose-dependence of the anti-allergic effects of beta-escin in the PCA mouse model. Beta-escin was applied at dosages of 1.0, 1.5, 2.0, 2.5 and 3.0 mg/kg 6 h prior challenge. Beta-escin significantly suppressed the extravasation of Evans blue into skin tissue in a dose-dependent manner (Figure 1B). The lowest tested dose of 1.0 mg/kg showed no effect on the PCA reaction yet. Starting at a concentration of 1.5 mg/kg of beta-escin, an inhibitory effect became detectable. The application of 2.0 and 2.5 mg/kg beta-escin strongly inhibited the PCA reactions, which resulted in statistically significant reductions by 21.8% and 44.8%, respectively, when compared to the vehicle-treated group. The strongest effect was seen with the dose of 3 mg/kg, which reduced the reaction by 55.3%.

These results clearly show that the anti-allergic effects of beta-escin are dose-dependent.

### Effects of beta-escin after different periods of pre-treatment in the murine PCA model

**Figure 2:**
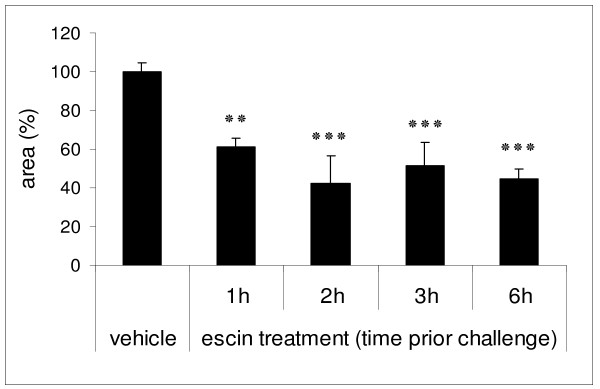
**Effects of beta-escin at different time points of pre-treatment in the murine PCA model**. SKH1-mice were injected with beta-escin (3 mg/kg) at different time points prior PCA induction as described in Fig. 1. For each mouse the mean of 6 spots was calculated. Based on these values the mean ± SEM was determined for 4 - 12 (escin) or 21 (vehicle) mice per treatment group and normalized to the vehicle control group. Significant differences between the treatment groups and the vehicle group are indicated with: ** p < 0.01 and *** p < 0.001.

To determine the influence of the pre-treatment period on the PCA reaction, mice were injected with 3 mg/kg beta-escin 1, 2, 3 or 6 h before induction of PCA (Figure 2). After pre-treatment for 1 h, beta-escin already inhibited the reaction by 39.1% compared to the vehicle group. The effect increased to 57.4% inhibition of the PCA reaction after pre-treatment for 2 h. At the 3 h and 6 h time points, the response was inhibited by 48.5% and 55.3%, respectively. Longer periods of pre-treatment (12 or 15 h) did not further improve the efficacy of beta-escin (data not shown). In conclusion, beta-escin significantly inhibited the PCA reaction at every tested time point in comparison to the negative control group. The course of this treatment response is consistent with the reported pharmacokinetic properties of beta-escin where a maximal serum level was reached after 2 h and the half-life was calculated to be 6 to 8 h [13].

This series of experiments in the PCA model strongly suggested that beta-escin is beneficial for the treatment of immediate type I hypersensitivity reactions and anaphylaxis. Therefore, it was of great interest to expand our studies to an additional model which reflects the late phase of an allergic response, such as a murine model of allergic airway inflammation.

### Beta-escin reduces the infiltration of total leukocytes and eosinophils into the airways in a murine model of allergic airway inflammation

**Figure 3:**
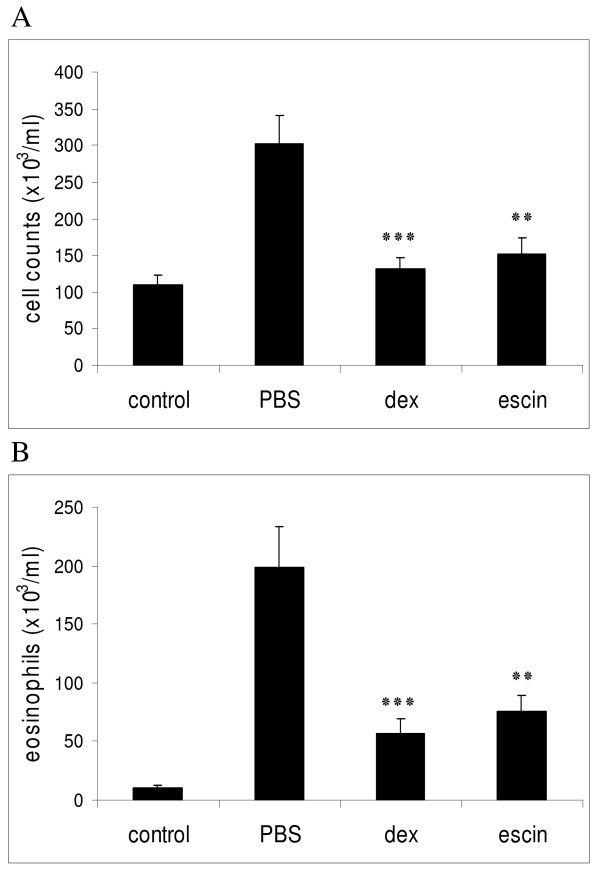
**Beta-escin reduces total cells and eosinophil recruitment in an OVA-induced model of allergic airway inflammation**. BALB/c mice were sensitized with OVA on days 0, 14 and 21. The aerosol challenges with OVA or PBS were performed twice a day on days 28 and 29. 40 h after the last aerosol challenge, the mice were sacrificed for the analyses of the airway inflammation of the lungs. Groups were treated as follows: control group = PBS-challenged, treated with PBS; PBS group = OVA-challenged, treated with PBS; dex group = OVA-challenged, treated with dexamethasone (1 mg/kg, once a day) and escin group = OVA-challenged, treated with beta-escin (3 mg/kg, twice a day). Groups were compared with respect to the total cell counts (A) and the number of eosinophils (B) in the BALFs. Each bar represents the mean of 13 - 21 mice from 4 independent, but identical experiments ± SEM out of a series of similar experiments. Significant differences of the dex and escin groups to the PBS group are indicated with: ** p < 0.01 and *** p < 0.001.

To determine whether beta-escin has effects on allergic airway inflammation, OVA-sensitized mice were challenged with aerosol containing either OVA antigen or the vehicle control PBS. Total cell infiltration, eosinophilia and Th2-cytokine secretion was then determined in the Broncho Alveolar Lavage Fluids (BALFs) of these mice. In the positive control group (PBS-treated mice), OVA challenge caused a marked increase of inflammatory cells in the BALF, especially eosinophils, which reflects the intensity of airway inflammation and the extent of cell infiltrates into the lungs. This was absent in the PBS-challenged negative control group (Figures 3A and 3B). In these experiments, dexamethasone was again used as a reference drug. A dosage of 1 mg/kg dexamethasone once a day was selected on the basis of its molecular mass (which is 2.8-fold less than of beta-escin, as discussed above), based on our own preliminary experiments, and previous reports [29,30]. This reference compound prevented cell infiltration into the airways by 56.6% at 40 h (Figure 3A).

Based on the results of the PCA model, a dose of 3 mg/kg beta-escin was administered twice daily to maintain the serum level of beta-escin during the challenge period. The number of total BAL cells was significantly decreased in the beta-escin-treated group in comparison to the PBS-treated group, resulting in a reduction by 49.5% at 40 h after the last challenge (Figure 3A). Moreover, both beta-escin and dexamethasone treatment significantly reduced the number of eosinophils in the BALF. Beta-escin treatment potently inhibited eosinophil infiltration into the airways by approximately 61.7% at 40 h (Figure 3B). Our findings indicate that beta-escin reduces airway inflammation and prevents eosinophil infiltration into the airways in this allergy model.

### Beta-escin reduces the release of asthma-associated cytokines in BALF

**Figure 4:**
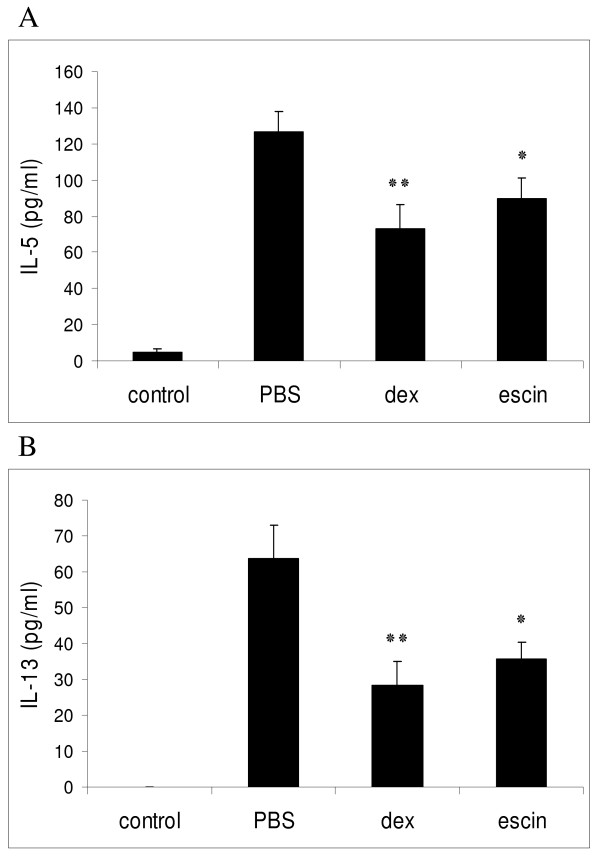
**Beta-escin reduces cytokine production in an OVA-induced model of allergic airway inflammation**. BAL fluid was collected 15 h after the last OVA-challenge from sensitized BALB/c mice of the 4 different treatment groups as described in Fig. 3. (A) IL-5 and (B) IL-13 levels in the BALF of individual mice from each group were determined by ELISA. Each bar represents the mean of 11 - 16 mice from 3 independent, but identical experiments ± SEM out of a series of similar experiments. Significant differences of the dex and escin groups to the PBS group are indicated with: * p < 0.05 and ** p < 0.01.

Th2-associated cytokines are typically released in allergic airway reactions. To evaluate the effects of beta-escin on cytokine secretion, the levels of IL-5 and IL-13 were determined in BALF supernatants of individual mice from each group. It was previously demonstrated in kinetic studies that the release of different types of cytokines into BALF of asthmatic mice peaks at different time-points. The maximal secretion ranged from as early as 2 - 4 h for TNF-alpha to 8 - 12 h, with a long lasting elevation, for IL-5 and IL-13 [31]. Based on this study we decided to perform the collection of BALF for the evaluation of IL-5 and IL-13 cytokines at 15 h after the last challenge. As shown in Figures 4A and 4B, IL-5 and IL-13 levels from OVA-challenged and PBS-treated mice of the positive control group were markedly increased compared to the negative control group (challenged and treated with PBS). The high concentration of IL-5 (126.6 ± 11.4 pg/ml) of the positive control group was significantly reduced by beta-escin treatment (90.0 ± 10.8 pg/ml), which was comparable to the reduction achieved by dexamethasone treatment (73.2 ± 12.8 pg/ml). Similarly, the IL-13 concentration was reduced to 35.8 ± 4.4 pg/ml by beta-escin treatment, which represented an inhibitory effect of 43.8% compared to the positive control group (63.7 ± 9.5 pg/ml). Again this effect was comparable to the reference compound, because dexamethasone reduced the IL-13 concentration to 28.2 ± 7.0 pg/ml (inhibition of 55.7%).

These results indicate that beta-escin successfully suppresses the secretion of Th2-cytokines into the airways of OVA-challenged mice.

### Beta-escin reduces the infiltration of inflammatory cells into lung tissue

**Figure 5:**
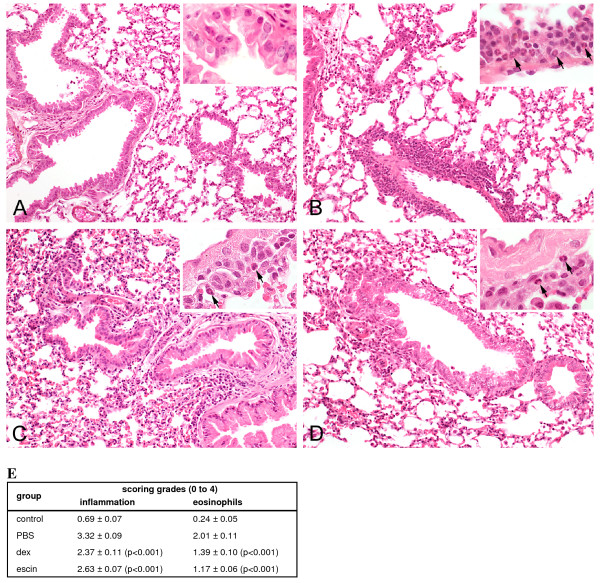
**Beta-escin reduces inflammation and eosinophilia in lungs of mice from the allergic airway inflammation model**. Representative photomicrographs in a 20 and 60 times magnification (inset) of H&E stained lung sections from 40 h after the last challenge show the lung parenchyma and the peribronchial and perivascular infiltration of inflammatory cells from (A) PBS-challenged mice treated with PBS (negative control); (B) OVA-challenged mice treated with PBS (= positive control); (C) OVA-challenged mice treated with dexamethasone (1 mg/kg, once a day) and (D) OVA-challenged mice treated with beta-escin (3 mg/kg, twice a day). The arrows indicate representative eosinophils in the infiltrate. (E) Semi-quantitative analyses of peribronchial and perivascular inflammatory cells and percentage of eosinophils in lung sections were performed with a scoring system. Each value represents the mean of 13 - 21 mice from 4 independent, but identical experiments ± SEM out of a series of similar experiments. Significant differences of the dex and escin groups to the PBS group are indicated in brackets.

To further examine the effects of beta-escin on lung pathology, histological analyses of lung sections were performed. Lung tissue was collected 40 h after the last aerosol challenge and analyzed by an experienced pathologist who was blinded to the sample identity to ensure an unbiased evaluation. To quantify the observed changes in lung tissue, inflammation and eosinophil scorings were calculated for each mouse sample from all 4 experimental groups, examples of which are presented in Figures 5A - D. In comparison to the negative control group, the lung histology from PBS-treated and OVA-challenged mice showed intense inflammatory cell infiltrates containing lymphocytes, neutrophils, macrophages and to a large extent eosinophils in the peribronchiolar and perivascular connective tissue (Figures 5A and 5B). Dexamethasone treatment attenuated the inflammatory cell infiltration in the lung tissue (Figure 5C). As expected, also beta-escin significantly reduced inflammation in lung tissue. In particular, the infiltration of eosinophils was suppressed compared to the PBS group (Figure 5D). Figure 5E summarizes the scoring for inflammation and eosinophilia for the individual treatment groups and highlights the statistical significance of the improvements by beta-escin or dexamethasone treatment.

These inhibitory effects of both drugs are in agreement with the determined cell types and numbers in the BALFs and confirm that beta-escin exerts an ameliorating effect on the inflammation in the airways and also in the lung tissue.

## Discussion

This report presents the first study of the effect of the natural triterpene saponin beta-escin on the early and late phases of type I hypersensitivity *in vivo*. Our results demonstrate that beta-escin strongly inhibits allergic reactions. A benchmark comparison indicates that beta-escin is comparable or even superior to dexamethasone, a standard reference compound for the evaluation of anti-allergic and anti-inflammatory drugs.

We first pinpointed the anti-allergic effect of beta-escin in the murine PCA model, where beta-escin potently suppressed the localized mast cell-mediated allergic reaction. The evaluation of a series of pre-treatment periods showed that the protective effect of beta-escin lasted at least for 6 h, which is consistent with published pharmacokinetic data [13]. Moreover, the PCA inhibition was strictly dose-dependent, which confirmed the pharmacologically relevant dose-response correlation of beta-escin with the reduction of symptoms.

In the model of acute allergic airway inflammation we could show that beta-escin effectively suppressed also the late phase allergic response. Beta-escin exerted beneficial effects on airway and lung inflammation by reducing leukocyte infiltration and eosinophilia in the lungs. In addition, it significantly decreased the release of Th2-specific cytokines into the BALF, as exemplified by IL-5 and IL-13 measurements.

Th2-cytokines play a pivotal role in the pathogenesis of asthma. The cytokine IL-5 initiates the terminal differentiation, proliferation, migration and activation of eosinophils [6,8]. IL-13 can also regulate the recruitment of eosinophils to the airways through its various effects on epithelial cells and smooth muscle cells [32]. Consistent with the reduction of these cytokines, the recruitment of inflammatory cells, especially of eosinophils, was markedly suppressed in the BALF by beta-escin treatment. The histo-pathological examination showed milder pathology, reduced inflammatory cell infiltrates and a lower density of eosinophils in the lungs of beta-escin-treated mice, which further supported our results from the BALF analysis. Eosinophils release a number of cytotoxic proteins, such as major basic protein, platelet activating factor and leukotrienes, which are responsible for the characteristic damage of the airway epithelium in allergic asthma [33,34]. The reduction of eosinophil recruitment to the pulmonary tissue by beta-escin is therefore pivotal to minimize tissue damage and/or improve recovery.

Airway inflammation constitutes a key symptom in the pathogenesis of asthma. Therefore, strategies which inhibit this initial phase may be regarded as a promising treatment approach [2,9,34]. Nonetheless, asthma is characterized as a chronic airway disease, which includes airway obstruction and remodelling of lung tissue. Nowadays, inhaled corticosteroids (ICS) are the primarily used agents for treating persistent asthma. Whereas several and sometimes serious side effects are observed for oral corticosteroids, ICS minimize systemic exposure and are typically well tolerated. However, a number of asthma patients are resistant to treatments with ICS or even to oral glucocorticoids [35]. This clearly indicates a medical need for the development of alternative anti-asthmatic drugs with good tolerability. Previously it was shown that extracts of Diplotropis ferruginea, Ganoderma tsugae and ginseng attenuate allergic airway inflammation in murine asthma models [29,36,37]. Ginseng for example was effective in resolving chronic histo-pathological changes in the lungs [29]. However, despite the low incidence of toxicity of ginseng, adverse reactions, such as insomnia, hypertension and diarrhoea have been reported [29].

For beta-escin, one case of occupational asthma related to beta-escin inhalation is described [38]. This patient was exposed to low-doses of beta-escin daily for 30 years and the onset of symptoms occurred 20 years after the initial exposure. While this case appears to be a singular incidence, it cautions that beta-escin may not be recommended for an aerosol inhalation therapy. Oral administration of beta-escin is a preferred and safe alternative, and it is not expected that oral treatment with beta-escin on clinical demand will cause or exacerbate asthma. Patients suffering from venous insufficiencies have been treated with beta-escin for more than 30 years, without a report of an increase in allergies.

Generally, beta-escin is considered to have a very low level of adverse reactions. A very low incidence of toxic effects has been observed in clinical trials using well-characterized preparations [13], which is highly encouraging for the further development of beta-escin towards an alternative to current allergy medications.

### Future perspective

We have shown that beta-escin is highly potent if applied by intraperitoneal administration. In view of a clinical application in humans in the future, further application routes, such as oral and intranasal, will be examined in models of allergy. An intranasal application may be suitable for the localized treatment of allergic rhinitis if a droplet size is generated that precludes aerosol inhalation to the lungs. A systemic treatment by oral application may be preferred for patients with asthma.

In order to investigate if a therapeutic application of beta-escin is equally effective for the control or reversion of pulmonary pathologies and the remodelling of lung tissue at a late stage of the disease, additional studies of long-term treatment with beta-escin in a chronic asthma model are planned. To receive more insight into the mode of action, further research is required to explain the influence of beta-escin on intracellular signaling cascades in mast cells, such as mitogen- or stress-activated protein kinase pathways, on calcium influx and on transcription factors such as NF-κB and NF-AT [3,39,40], and on the stabilization of membranes. A very recent study showed that beta-escin can impair the NF-κB-signaling cascade in cancer cell lines [41]. Considering the key role of NF-κB in immune responses and inflammation, it is an intriguing possibility that beta-escin inhibits allergic reactions by interfering with NF-κB-signaling in a similar manner. Elucidating the molecular mode of action of beta-escin will support the further development of beta-escin as anti-allergic medication.

## Conclusions

The results of our studies indicate that beta-escin provides a highly effective treatment against early as well as late phase allergic responses in two different *in vivo *models for type I hypersensitivity reactions. Considering the beneficial effect seen in our experiments and the excellent tolerability and safety profiles proven in several animal models and human clinical studies, beta-escin has an exciting therapeutic potential for the treatment of allergic inflammatory diseases such as allergic rhinitis and allergic asthma in humans. Therefore, clinical trials are planned to confirm our observations also in patients who suffer from allergies.

## Methods

### Animals

Specific pathogen-free SKH1-mice (strain 313, hairless, Charles River, Germany), were used for the PCA model, and BALB/c mice (Harlan, Italy) for the asthma model. All mice were maintained under standard laboratory conditions in the animal facility of the Veterinary University Vienna. All experimental procedures were discussed and approved by the institutional ethics committee and performed under the Austrian experimental animal license numbers 68.205/0134-C/GT/2007 and 68.205/0206-II/10b/2008.

### Test and reference compounds

Beta-escin (CAS No. 6805-41-0; Euro OTC, Germany, and Nobilus Ent, Poland) and water-soluble dexamethasone (CAS No. 50-02-2; Sigma-Aldrich, Austria) were dissolved in 0.9% sodium chloride (NaCl; Mayrhofer, Austria) or in phosphate buffered saline (PBS; PAA, Austria) in indicated concentrations for intraperitoneal (i.p.) administration.

### Passive Cutaneous Anaphylaxis model

Hairless SKH1-mice, female, 10 weeks old, were pre-treated i.p. with beta-escin, dexamethasone (3 mg/kg) or vehicle (NaCl or PBS, 10 ml/kg) at different dosages and time points prior induction of a PCA reaction as indicated in the text and figure legends. To initiate the PCA reaction, 2.5 μg anti-TNP IgE (BioLegend, Austria) diluted in 10 μl of 0.9% NaCl solution was intradermally injected into the dorsal skin at 6 independent spots per animal. Fifteen minutes after sensitization, the mice were challenged by intravenous injection of 0.1 ml 0.9% NaCl containing 50 μg of DNP-BSA (Calbiochem, Austria) and 1% Evans blue (Sigma-Aldrich, Austria). Mice were sacrificed 15 min after DNP-BSA injection and their dorsal skin was removed for measurement of the extension of the blue stained skin areas by analysis of digital photos using ImageJ software (1.39 d version; NIH; http://rsb.info.nih.gov/ij/) [25]. The effects of beta-escin and dexamethasone were expressed in percent of mean values of the vehicle control samples.

### Induction of allergic airway inflammation and treatment schedule

Female BALB/c mice, 6 - 8 weeks old, were divided into four groups (n = 10 - 13 animals per group). All mice were immunized on days 0, 14 and 21 by i.p. injections of 10 μg of OVA (Grade V, Sigma-Aldrich, Austria) and 2 mg of Al(OH)_3 _(Pierce, USA) suspended in 0.2 ml of PBS. On days 28 and 29 after the initial immunization, animals were challenged for 60 min twice daily with 1% OVA dissolved in PBS and aerosolized by an ultrasonic nebulizer (Kendall Aerodyne 18605, Covidien, Austria). Negative control mice were treated in parallel with PBS aerosol only. 15 or 40 h after the last challenge (days 30 and 31), the mice were sacrificed, and allergic airway inflammation was characterized. The 4 treatment groups were as follows: control group (negative control group) = challenged with PBS and treated with PBS (10 ml/kg, twice a day, i.p.), PBS group (positive control group) = challenged with OVA and treated with PBS as vehicle control (10 ml/kg, twice a day, i.p.), beta-escin group = challenged with OVA and treated with beta-escin (3 mg/kg, twice a day, i.p.), and dex group = challenged with OVA and treated with dexamethasone (1 mg/kg, once a day, i.p.). The treatments started on day 27 and ended on day 30 for the assessment at 15 h and on day 31 for the assessment at 40 h after the last aerosol challenge.

The presence of OVA-specific IgE antibodies in the sera of all OVA-immunized mice was verified by an OVA-ELISA kit (MD Biosciences, USA) to ensure that the observed differences in the treatment groups were indeed specific to the respective treatments and not caused by lack of OVA-specific reactions due to a failed immunization.

### BAL for determination of cell counts, leukocyte differentiation and cytokine levels

To perform BAL, animals were anaesthetized and a 23-gauge cannula was inserted into the trachea. 1 ml of ice-cold PBS was instilled with a syringe and recovered by gentle aspiration. The recovered BALF was centrifuged (150 × g at 4°C for 5 minutes) and the supernatant was recovered for the determination of cytokines in the BALF. The cell pellet was resuspended in 0.5 ml ice-cold PBS. The total and differential cell counts were determined by the automatic cell counter ADVIA 120 (Bayer Diagnostics, Austria). Cytokines in BALF supernatants were measured by ELISA for IL-5 (BioLegend, Austria) and IL-13 (BenderMedSystems, Austria) according to the manufacturer's instructions. The detection limit for IL-5 and IL-13 was 7.8 pg/ml.

### Histological assessment of inflammation and eosinophils in lung tissue

Mice were sacrificed 40 h after the last aerosol challenge. Following the collection of blood and BAL, the lung tissues were removed, fixed in 7% neutral-buffered formalin solution, embedded in paraffin, cut in 3 μm sections, and stained with Hematoxylin & Eosin (H&E) for examining the cell infiltration.

Lung tissues were evaluated by an experienced pathologist who had been blinded to the treatment groups. The degree of peribronchiolar and perivascular inflammation was evaluated semi-quantitatively by a scoring system which was defined by the number of peribronchiolar inflammatory cells: 0 = no cells; 1 = a few cells; 2 = a ring of cells 1 cell layer deep; 3 = a ring of cells 2-4 cells deep; 4 = a ring of cells >4 cells deep [42]. The percentage of eosinophils from the inflammatory cells was determined and categorized from 0 to 4: 0 = no eosinophils; 1 = 25%, 2 = 25 - 50%; 3 = 50 - 75% and 4 = > 75% of inflammatory cells.

### Statistical analysis

Data were expressed as the mean ± standard error of the mean (SEM), checked for normal distribution and their statistical significance was determined by Student's two-tailed t-test for independent means, using SPSS (PASW Statistics 17.0). P < 0.05 was considered to be statistically significant.

## Abbreviations

BALF: bronchoalveolar lavage fluid; ICS: inhaled corticosteroids; IgE: Immunglobulin E; i.p.: intraperitoneal; OVA: Ovalbumin; PCA: Passive Cutaneous Anaphylaxis

## Competing interests

The authors CM, AG, EP and PD are employed by Marinomed Biotechnologie GmbH. The author HU is co-founder of Marinomed. AG and EP are inventors on patent # WO 2008015007 held by Marinomed Biotechnologie GmbH that relates to the content of the manuscript. Marinomed Biotechnologie GmbH is financing the processing charge of this manuscript.

## Authors' contributions

AG, EP, IL and PD participated in design and interpretation of the experiments. AU performed the scoring and assessment of the lung histopathology in the model of allergic airway inflammation. CM conducted the *in vitro *assays and was engaged in the establishment of the PCA model. IL and PD carried out the *in vivo *experiments and assessments and drafted the manuscript. AG, EP, IL, PD and HU contributed to experimental designs of the study and writing of the manuscript. All authors read and approved of the final manuscript.
